# CD163+ macrophage density in perimysial connective tissue associated with prognosis in IMNM

**DOI:** 10.1002/acn3.52065

**Published:** 2024-04-23

**Authors:** Hui Sun, Zi‐Yi Wang, Ye Han, Xiao‐Jing Wei, Yong‐Chun Wang, Xue‐Fan Yu

**Affiliations:** ^1^ Department of Neurology and Neuroscience Center The First Hospital of Jilin University, Jilin University Changchun China; ^2^ Center for Rare Diseases The First Hospital of Jilin University, Jilin University Changchun China

## Abstract

**Objective:**

The pathological features of immune‐mediated necrotizing myopathy (IMNM) are dominated by the infiltration of macrophages. We aimed to perform a histopathologic semiquantitative analysis to investigate the relationship between macrophage markers and prognosis.

**Methods:**

Semiquantitative analysis of histologic features was performed in 62 samples of IMNM. Independent risk factors were identified through univariate and multivariate regression analysis. Cluster analysis was performed using the partitioning around the medoids (PAM) method. Decision tree modeling was utilized to efficiently determine cluster labels for IMNM patients. The validity of the developmental cohort was assessed by accuracy in comparison with the validation cohort.

**Results:**

The most enriched groups in patients with IMNM were macrophages expressing CD206 and CD163. In the multivariate logistic regression model, the high density of CD163+ macrophages in perimysial connective tissue increased the risk of unfavorable prognosis (*p* = 0.025, OR = 1.463, 95% CI: 1.049–2.041). In cluster analysis, patients in Cluster 1, with lower CD163+ macrophage density and inflammatory burden, had a more favorable prognosis. Conversely, patients in Cluster 3, which were enriched for CD163+ macrophages in the perimysial connective tissue, had the most severe clinical features and the worst prognosis. Correlations were found between the density of CD163+ macrophages in connective tissue and symptom duration (*R* ^2^ = 0.166, *p* < 0.001), dysphagia (*p* = 0.004), cardiac involvement (*p* = 0.021), CK (*R* ^2^ = 0.067, *p* = 0.042), CRP (*R* ^2^ = 0.117, *p* < 0.001), and ESR (*R* ^2^ = 0.171, *p* < 0.001).

**Conclusion:**

The density of CD163+ macrophages in perimysial connective tissue may serve as a potential marker for the prediction of IMNM prognosis.

## Introduction

Immune‐mediated necrotizing myopathy (IMNM) is a rare autoimmune disorder and one of the independent subtypes of idiopathic inflammatory myopathies (IIMs). It is characterized by dysphagia, limb weakness, and elevated serum creatine kinase (CK) levels.^1^ Currently, IMNM has been categorized into three subtypes by previous studies: anti‐signal recognition particles (anti‐SRP), anti‐3‐hydroxy3‐methylglutaryl‐coA reductase (anti‐HMGCR), and seronegative IMNM.^2^ Due to the disabling and refractory nature of IMNM, achieving disease control with steroid monotherapy alone is more restricted. Furthermore, over a 4‐year follow‐up, Johns Hopkins observed that only 50% of SRP‐IMNM patients achieved near‐normal muscle strength with a combination of conventional steroids and standard immunosuppressants.^3^ Some patients continued to have a persistent elevation of serum CK levels despite continuous treatment.^3^ Zhao et al. demonstrated that high expression of B‐cell activating factor receptors in myofibrils is a risk factor for poor response to treatment in anti‐SRP‐IMNM.^4^ This finding suggests that patients with IMNM who are refractory to standard immunosuppressive therapies may benefit from lymphocyte clearance therapies such as rituximab or belimumab.^5^, ^6^ However, research is lacking on risk factors associated with insensitivity to immunotherapy or relapse in individuals with IMNM.

Histologic features of IMNM include marked myofibrillar degeneration, necrosis, and macrophage infiltration, with lymphocyte invasion in some patients.^7^, ^8^ Macrophages, crucial components of the immune system, have been recognized for their significant involvement in various diseases, including systemic lupus erythematosus (SLE),^9^ rheumatoid arthritis,^10^ and vasculitis.^11^ Numerous studies have shown that macrophages possess the ability to differentiate into distinct subtypes with divergent functions, in the development, progression, and alleviation of immunoinflammatory diseases.^12^ Currently, macrophages can be classified into two groups: M1 macrophages, which have pro‐inflammatory functions and express the marker inducible nitric oxide synthase (iNOS), and M2 macrophages, which predominantly express the markers CD206+ and CD163+, involved in anti‐inflammatory and fibrotic repair.^9^ In histology, the distribution and density of macrophage polarization mechanistically impact complement deposition and endomyocardial capillary density in muscle fibers of patients with IMNM.^13^ The balance between the activity of the M1 and M2 macrophage subtype accounts for the different pathological features observed in patients with IMNM.^13^ However, the distribution and density of macrophage markers have not been analyzed in large samples of IMNM. Thus, our study aims to investigate the role of macrophage markers in the prognosis of IMNM using the sample bank from the Neuromuscular Disease Center in Northeast China.

## Methods

### Ethical support

The study adhered to the principles of the Declaration of Helsinki and received approval from the Ethics Committee of the First Hospital of Jilin University (Approval number: AF‐IRB‐032‐06). Muscle samples were donated by patients who provided informed written consent. Since the retrospective nature of the study and the anonymity maintained in the data, the need for obtaining informed consent was waived.

### Population

Patients diagnosed with IMNM according to the 119th^7^ and 224th^2^ European Neuromuscular Centre criteria between January 2015 and December 2021 at the First Hospital of Jilin University were included. The enrolled patients underwent retrospective confirmation by two experienced neurologists (HS and XY). Exclusion criteria included patients under 18 years of age, with immunotherapy, and those with incomplete clinical data or follow‐up.

### Data collection

The study examined the electronic medical records of patients and rare disease registries. It recorded demographic characteristics such as age and sex, along with clinical manifestations including cervical flexion weakness, severe myasthenia, myalgia, dysphagia, amyotrophy, and weight loss. Laboratory findings, including lymphocyte, neutrophil, and platelet counts, Complement 3 (C3), Complement 4 (C4), CK, erythrocyte sedimentation rate (ESR), C‐reactive protein (CRP), and antinuclear antibody levels (ANA), were collected. The serum for laboratory tests was obtained in the morning and without immunotherapy. Severe myasthenia was defined as a mRS score >2 at the initial diagnosis. Respiratory, endocrine, and cardiology specialists collaborated to identify instances of interstitial lung disease (ILD), thyroid involvement, or cardiac complications. Thyroid involvement was defined as thyroid cancer or thyroid dysfunction. Cardiac involvement was defined as myocarditis, pericardial effusion, cardiomyopathy, arrhythmia, or coronary artery disease.^14^ Tumors identified within 3 years before or after the diagnosis of IMNM were classified as myositis‐associated tumors.^15^ Myositis‐specific antibodies (MSA) were detected using Nanjing Simcere or Beijing Dian Diagnostics. Of the total number of patients, 57 (91.9%) received more than one type of immunotherapy. The median follow‐up period was 33 months (IQR, 21–45 months). The administered immunotherapeutic drugs included glucocorticoid (*n* = 58), methyl‐macrolide (*n* = 29), tacrolimus (*n* = 7), methotrexate (*n* = 19), intravenous immunoglobulin (*n* = 3), and other immunosuppressants (*n* = 7).

### Histological semi‐quantification

Muscle samples were stored at −80°C before being cut into 10 μm sections for histologic and immunohistochemical (IHC) staining. The number of positive cells was counted in 10 randomly selected fields of view (original magnification 200) per section, and the mean ratio of positive cells to the area of the region was recorded as the density per variable. The categorical variables used for C5b‐9 deposition are referenced in Olivier et al. (Table S1).^16^ QuPath, an open‐source software (https://qupath.github.io/), was utilized to quantify the target area and DAB‐positive cells and to calculate the mean cell density. Necrotic muscle fibers were identified as hyalinized or phagocytosed muscle fibers in hematoxylin and eosin staining while regenerating muscle fibers were characterized by staining positive for CD56 staining (1:2000, 14255‐1‐AP, Proteintech).^17^, ^18^ The densities of CD3 (1:200, ab16669, Abcam), CD4 (1:200, ab133616, Abcam), CD20 (1:200, ab78237, Abcam) lymphocytes, CD68 (1:200, ab213363, Abcam) macrophage, and C5b‐9 (1:150, ab55811, Abcam) were measured in the assessment. Especially, the density of the macrophage subtype was assessed by infiltrating the endomysial, perivascular, and perimysial connective tissue (Fig. S1). We utilized primary antibody markers to assess macrophage expression, specifically iNOS (1:1000, 22226‐1‐AP, Proteintech), CD206 (1:1000, 60143‐1‐IG, Proteintech), and CD163 (1:1000, 16646‐1‐AP, Proteintech). Two experienced neuromuscular pathologists (HS and YH) independently scored anonymized samples, and the scores were averaged. The results were analyzed using the Kappa consistency test (Kappa = 0.617–0.842, *p* < 0.05) by two observers. Variables with *p* values exceeding 0.05 for differences were recalculated after consensus review.

### Outcome

The study defined the outcome as a favorable prognosis for patients with an mRS score of 0–2 after 12 months of immunotherapy. Patients with an mRS score of 3–5 or those who were refractory were included in the unfavorable prognosis group. Refractory was defined as an increase in CK levels above the patient's baseline, retreatment with glucocorticoids, or modification of the immunotherapy regimen due to worsening muscle strength.^4^

### Cluster and decision tree

The partitioning around the medium (PAM) cluster analysis enables the inclusion of both continuous and categorical variables. In this analysis, we grouped patients with characteristics using 18 pathologic variables (Table S1). To eliminate the effects due to different units or magnitudes, we used *z*‐scores to ensure consistency [*z*‐score = (*xi* − mean(*x*))/SD(*x*)]. The optimal number of clusters for the data was determined using the average silhouette method. Silhouettes were calculated for different *k* values, and the *k* value with the highest mean silhouette was chosen as the number of clusters. The clustering results were evaluated using the silhouette coefficient.

To assign patients to clusters, the categorical regression decision tree was utilized to identify variables associated with each cluster. “High” densities were defined as continuous variables with values greater than the 75th percentile of the total value. The number of end nodes in the simplified decision tree model was determined by the unsupervised machine learning method, cost complexity (CP).

### Statistical analysis

Non‐normally distributed continuous variables were described using the median and interquartile range (IQR). Categorical data were presented as ratios and percentages. Continuous variables were compared using either the Mann–Whitney *U* or Kruskal–Wallis test, and categorical variables were compared using either Fisher's exact test or the chi‐squared test. Correlation analysis was conducted using Pearson's correlation coefficient test. Risk factors for an unfavorable prognosis in patients with IMNM were identified using multivariate logistic regression. Statistical significance was determined for *p* values <0.05. Statistical analyses and graphical presentations were performed using GraphPad Prism (version 9.5.0), R (version 4.2.2, packages “Cluster” and “rpart”) and SPSS (version 26.0) software.

## Results

### Demographic and characteristics

Three samples (*n* = 3) were excluded due to ineligibility, as well as variables with more than 10% missing data, such as MSA antibody titers, electromyography, and serum ferritin. A total of 62 samples, 18 pathological variables, and 26 clinical parameters were available for statistical analysis. The majority of patients were female (66.1%), with a median age of 52 years (IQR 43–60 years), and a median symptom duration of 5 months (IQR 3–7 months) (Table 1).

**Table 1:** Comparison of clinical characteristics in different prognoses.

Variables	All (*n* = 62)	Unfavorable (*n* = 22)	Favorable (*n* = 40)	*p* value
Demographic data				
Age	52 (43, 60)	51 (41, 57)	52 (43, 61)	0.606
Female	41 (66.1)	16 (72.7)	25 (62.5)	0.416
Male	21 (33.9)	6 (27.2)	15 (37.5)	
Symptom duration (months)	5 (3, 7)	7 (5, 9)	5 (2, 6)	<0.001
Clinical features				
Cervical flexion weakness	26 (41.9)	12 (54.5)	14 (35.0)	0.136
Severe myasthenia	32 (51.6)	20 (90.9)	12 (30.0)	0.732
Myalgia	19 (30.6)	7 (31.8)	12 (30.0)	0.882
Dysphagia	11 (17.7)	4 (18.1)	7 (17.5)	0.031
Amyotrophy	10 (16.1)	5 (22.7)	5 (12.5)	0.295
Weight loss	15 (24.2)	8 (36.3)	7 (17.5)	0.466
Laboratory tests (IQR)				
Lymphocyte	1.49 (0.99, 2.12)	1.59 (0.87, 2.06)	1.45 (1.04, 2.29)	0.435
Neutrophil	4.76 (3.72, 6.55)	5.02 (4.01, 6.83)	4.50 (3.17, 6.37)	0.195
Platelet	246 (200, 280)	264 (221, 329)	223 (194, 275)	0.056
Complement 3	1.09 (0.96, 1.26)	1.10 (0.94, 1.25)	1.09 (0.97, 1.26)	0.991
Complement 4	0.24 (0.20, 0.29)	0.22 (0.17, 0.28)	0.24 (0.20, 0.32)	0.546
Creatine kinase	2037 (704, 5890)	3865 (1181, 7359)	1505 (626, 4931)	0.048
ESR	34.5 (19.0, 52.3)	47.00 (28.25, 68.26)	28.48 (12.81, 41.50)	0.005
C‐reactive protein	11.30 (3.28, 23.56)	15.55 (12.32, 28.97)	6.13 (3.17, 19.14)	0.001
MSA				0.878
SRP	28 (45.2)	10 (45.5)	18 (45.0)	
HMGCR	19 (30.6)	6 (27.3)	13 (32.5)	
Seronegative	15 (24.2)	6 (27.3)	9 (22.5)	
Anti‐Ro‐52 antibody	24 (38.7)	13 (59.1)	9 (22.5)	0.792
ANA	40 (64.5)	17 (77.3)	23 (57.5)	0.119
Complications				
Thyroid	15 (24.2)	5 (22.7)	10 (25.0)	0.842
Tumor	17 (27.4)	6 (27.3)	11 (27.5)	0.985
Cardiac involvement	19 (30.6)	9 (40.9)	10 (25.0)	0.061
Interstitial lung disease	14 (22.6)	10 (45.5)	4 (10.0)	0.539

Values are expressed as a percentage (%) or interquartile range.

ANA, antinuclear antibody; ESR, erythrocyte sedimentation rate.

**Figure 1:**
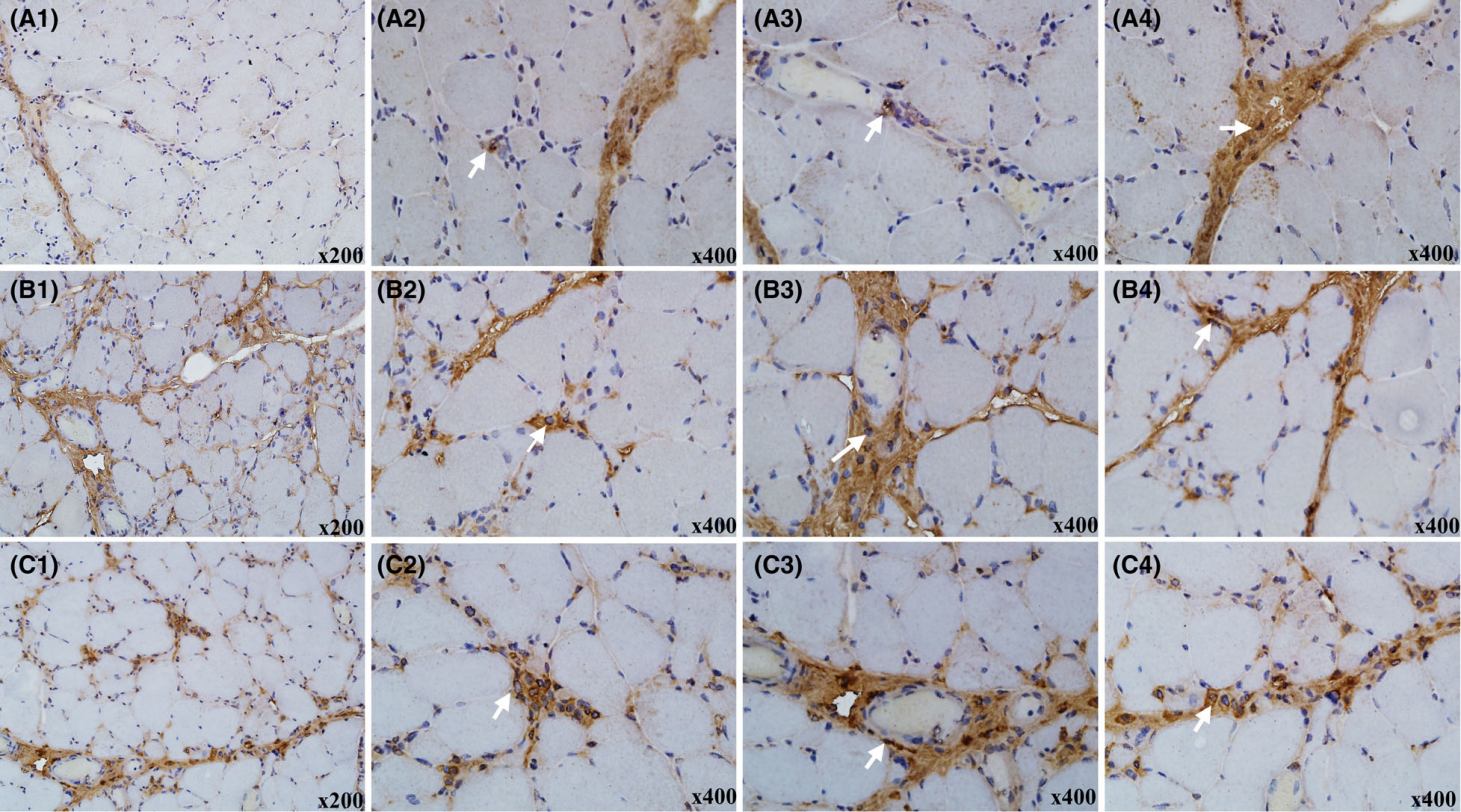
Macrophage markers distribution in IMNM patients. (A1) iNOS+ macrophages were found sparsely distributed in the endomysial (A2), perivascular (A3), and perimysial connective tissue (A4) (white arrow). (B1) CD206+ macrophages were widely distributed in the endomysial (B2), perivascular (B3), and perimysial connective tissue (B4). (C1) CD163+ macrophages were abundantly distributed in endomysial (C2), perivascular (C3), and perimysial connective tissue (C4).

Histologically, there was a low infiltration of iNOS+ macrophages, primarily involved in phagocytosis of degenerated or necrotic myofibers. A small amount of these macrophages was distributed in the vascular or connective tissue. Of the iNOS+ macrophages, 64.5% (*n* = 40) were distributed in the perivascular region, 100% (*n* = 62) in endomysial region, and 74.2% (*n* = 46) in perimysial connective tissue (Fig. 1). In contrast, the pathological distribution revealed that macrophages enriched for CD163+ and CD206+ expression were mainly located in the endomysial and perimysial connective tissues. Semiquantitative data of CD206+ macrophages were counted in all contiguous sections distributed in perivascular, endomysial, and perimysial connective tissues. The positivity rates were 69.4% (*n* = 43), 100.0% (*n* = 62), and 74.2% (*n* = 46), respectively. Additionally, CD163+ was the most frequent macrophage marker in IMNM. Macrophage CD163+ expression was detected positively in the perivascular region of 91.9% (*n* = 57) patients, as well as in the endomysial and perimysial connective tissues of all patients.

### Independent risk factors in logistic regression

Out of the 62 patients, 22 were classified as having an unfavorable prognosis. According to the univariate analysis, patients with clinical features including symptom duration, dysphagia, CK, ESR, and CRP levels increased the risk of unfavorable prognosis (Table 1).

**Figure 2:**
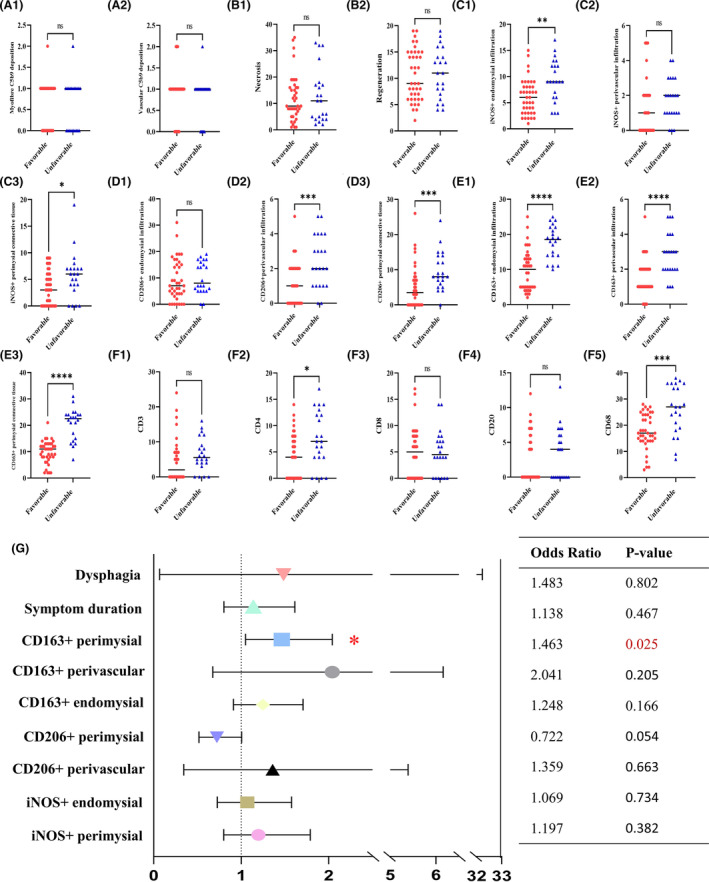
Identification of independent risk factors for unfavorable prognosis. There was no statistical significance of C5b9 deposition in endomysial (A1) or perivascular (A2) and the density of muscle fiber necrosis (B1) or regeneration (B2). The iNOS+ density was statistical differences in the endomysial (C1, 10^−1^ mm^2^) and perimysial connective tissue (C3, 10^−2^ mm^2^) rather than perivascular region (C2). CD206+ density was significantly higher in the perivascular (D2) and perimysial connective tissue (D3) in the unfavorable prognosis cohort, except in the endomysial region (D1). In the unfavorable cohort, the density of CD163+ macrophages was relatively higher (E1–E3). No statistically significant differences were observed in densities of CD3, CD8, CD20, and CD68 cells, except for CD4+ cell infiltration (F1–F5, 10^−1^ mm^2^). (G) Multivariable logistic regression analysis identified perimysial connective tissue CD163+ macrophage density as an independent risk factor. *p* values were calculated using the Mann–Whitney *U* test. *p < 0.05, **p < 0.01, ***p < 0.001, ****p < 0.0001.

In the perimysial connective tissue, macrophages positive for iNOS, CD206, and CD163 were significantly associated with prognostic differences (Fig. 2). The densities of iNOS+ and CD163+ macrophages in endomysial infiltration exhibited statistical significance (*p* < 0.001). Semi‐quantification of macrophage markers revealed increased densities of CD163+ and CD206+ macrophages in the perivascular region of the unfavorable group. CD4+ cell infiltration showed slight significance (*p* < 0.05), and there was no evidence of cytotoxicity (Fig. 2F1–F5). Myofiber necrosis, regeneration frequency, and C5b‐9 deposition were not statistically different in the two prognostic cohorts.

The multivariate regression included symptom duration and dysphagia as known risk factors. Furthermore, the model confirmed that the density of CD163+ macrophages in the perimysial connective tissue was an independent risk factor for an unfavorable prognosis (*p* = 0.025, OR = 1.463, 95% CI: 1.049–2.041, Fig. 2G and Table S2).

### Clustering according to macrophage and pathological characteristics

**Figure 3:**
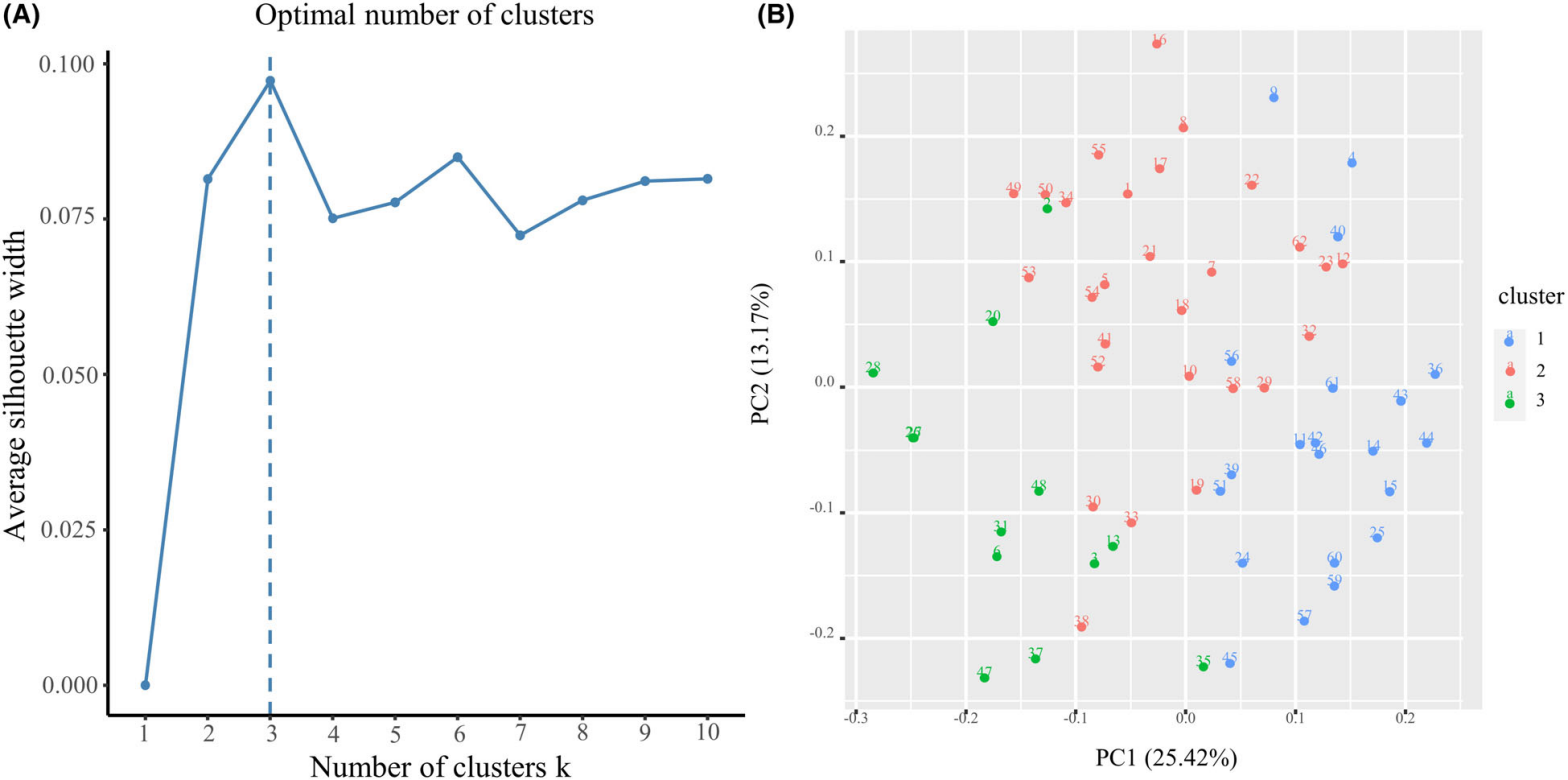
Patient clustering for three different clusters was determined using the silhouette method. (A) The average silhouette method was employed to calculate the optimal number of clusters (*k*‐value), resulting in a determination of 3 when the *y*‐axis observation reached its maximum (peak). (B) The patients were clustered using the partitioning around the medium method, and the results were visualized as a scatter plot using principal component analysis with two principal dimensions.

Unsupervised cluster analysis was conducted on the samples based on the semiquantitative assessment of macrophages and other histologic variables (Table S1). The silhouette method determined that the optimal number of clusters was 3 (Fig. 3A). Subsequently, cluster labels were assigned to each patient using the PAM method, and clustering was visualized by principal component analysis (Fig. 3B). The study found that 33.9% of patients were in Cluster 1 (*n* = 21), 45.2% were in Cluster 2 (*n* = 28), and 21.0% were in Cluster 3 (*n* = 13).

**Figure 4:**
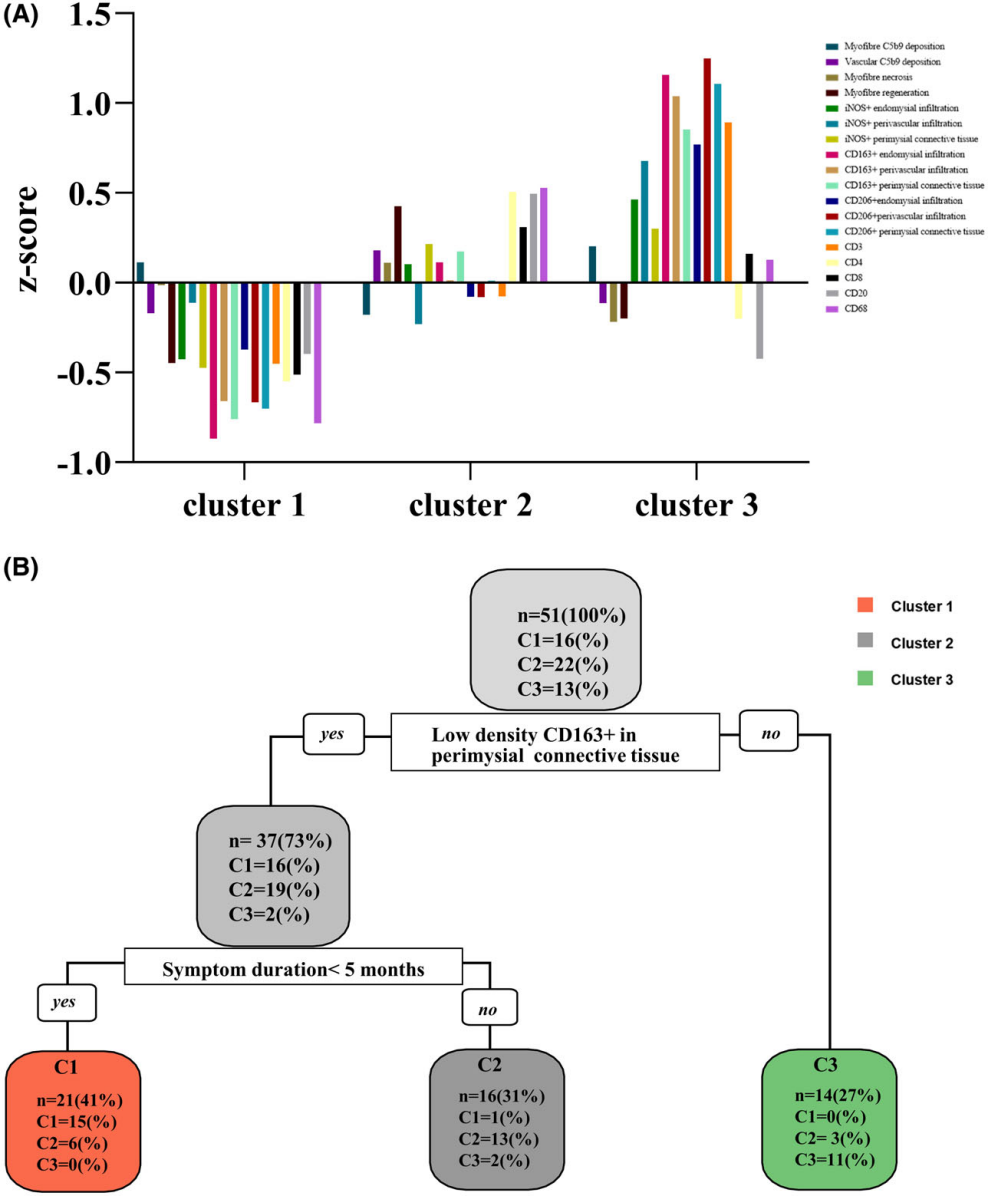
Pruned decision trees constructed using the development cohort. (A) The histograms represented the *Z*‐scores for each pathological variable in three clusters. (B) To construct a minimum‐error pruned decision tree, two critical variables were selected: low density of CD163+ macrophages in perimysial connective tissue, and 5 months of symptom duration.

Compared to Clusters 1 and 2, the density of CD163+ macrophages in perimysial connective tissue was significantly higher in Cluster 3 (*p* < 0.001, *p* = 0.039, Kruskal–Wallis test). Additionally, the three clusters presented different clinical parameters (Figs. 4A and S2). The median duration of symptoms in Cluster 1 was 4 months (IQR, 2.0 to 5.5, Table 2). Five patients experienced severe myasthenia, with cardiac involvement accounting for 9.5%. Serum C4 levels were higher in comparison with the other clusters, while ESR and CRP levels were lowest (*p* = 0.010, *p* = 0.008, Kruskal–Wallis test). Patients in Cluster 2 had intermediate disease duration, C4, ESR, and CRP levels, except for serum C3 levels, which were significantly higher than those in the other groups. In contrast, patients in Group 3 had the lowest serum C4 levels and the highest ESR and CRP levels. Additionally, a higher percentage of patients in Cluster 3 had cardiac involvement (*p* < 0.001). This cohort presented with a prolonged symptom duration, severe histological and clinical symptoms. The distribution of unfavorable prognosis among the three clusters was significantly different (*p* < 0.001). Patients in Cluster 3 had the worst recovery or response to immunotherapy.

**Table 2:** IMNM patients were classified into 3 groups using cluster analysis.

Variables	Cluster 1 (*n* = 21)	Cluster 2 (*n* = 28)	Cluster 3 (*n* = 13)	*p* value
Demographic data				
Age	47 (44, 58)	56 (45, 65)	48 (38, 53)	0.024
Female	13 (61.9)	21 (75.0)	7 (53.8)	0.363
Male	8 (38.1)	7 (0.25)	6 (46.2)	
Symptom duration (months)	4.0 (2.0, 5.5)	5.0 (4.0, 6.8)	6.0 (7.0, 12.0)	0.001
Clinical features				
Cervical flexion weakness	7 (33.3)	11 (39.3)	8 (61.5)	0.250
Severe myasthenia	5 (23.8)	14 (50.0)	13 (100.0)	<0.001
Myalgia	7 (33.3)	10 (35.7)	2 (15.4)	0.400
Dysphagia	2 (9.5)	4 (14.3)	5 (38.5)	0.081
Amyotrophy	2 (9.5)	6 (21.4)	2 (16.7)	0.532
Weight loss	6 (28.6)	5 (17.9)	4 (30.8)	0.566
Laboratory tests (IQR)				
Lymphocyte	2.03 (1.21, 2.50)	1.34 (0.90, 1.95)	1.21 (0.85, 2.12)	0.069
Neutrophil	4.53 (3.69, 6.39)	4.21 (3.19, 6.08)	4.79 (4.06, 7.18)	0.080
Platelet	223 (192, 271)	253 (192, 291)	275 (219, 359)	0.108
Complement 3	1.11 (1.06, 1.27)	1.14 (0.98, 1.29)	0.96 (0.80, 1.08)	0.029
Complement 4	0.24 (0.20, 0.33)	0.21 (0.18, 0.31)	0.20 (0.14, 0.26)	0.038
Creatine kinase	1926 (670, 5226)	1364 (808, 4379)	3279 (1253, 6820)	0.211
ESR	26.0 (13.1, 35.0)	32.5 (20.0, 50.4)	47.0 (30.7, 70.0)	0.010
C‐reactive protein	5.59 (3.19, 11.28)	12.66 (3.61, 27.38)	23.22 (13.40, 34.50)	0.008
MSA				
SRP	9 (42.9)	14 (50.0)	5 (21.5)	0.837
HMGCR	8 (38.1)	7 (25.0)	4 (30.8)	
Seronegative	4 (19.0)	7 (25.0)	4 (30.8)	
Anti‐Ro‐52 antibody	8 (38.1)	12 (42.9)	4 (30.8)	0.749
ANA	12 (57.1)	18 (64.3)	10 (76.9)	0.503
Complications				
Thyroid	5 (23.8)	7 (25.0)	3 (23.1)	0.990
Tumor	7 (33.3)	7 (25.0)	3 (23.1)	0.750
Cardiac involvement	2 (9.5)	8 (28.6)	9 (69.2)	0.001
Interstitial lung disease	5 (23.8)	8 (28.6)	1 (7.9)	0.326
Outcome				
Unfavorable prognosis	1 (4.8)	11 (39.3)	10 (76.9)	<0.001

Values are expressed as a percentage (%) or interquartile range.

ANA, antinuclear antibody; ESR, erythrocyte sedimentation rate.

### Construction and validation of decision tree

The predictive model was constructed for clusters using a simplified classification. The development cohort consisted of 51 patients diagnosed with IMNM between January 2015 and December 2018 at our institution. Variables for the tree nodes were selected based on univariate analyses and clinical relevance. To simplify the model, the CP method was used to determine the optimal decision variables (*n* = 2, Figs. S3 and S4). The decision tree was developed using two variables from a cohort of 51 patients: low perimysial connective tissue CD163+ infiltration (17 cells/10^−2^ mm^2^) and symptom duration (5 months) (cross‐validation = 50, Fig. 4B). An additional 11 IMNM patients diagnosed between January 2019 and December 2021 were used for external validation, confirming the decision tree's generalizability. The patients in the validation cohort were divided into three clusters, as described in PAM methods. We demonstrated the validity of using simple variables to group patients into clusters with accuracy. The development cohort achieved a predictive accuracy of 76.4%, while the validation cohort achieved an accuracy of 72.7% (Fig. S4 and Table S3).

### Additional clinical significance

**Figure 5:**
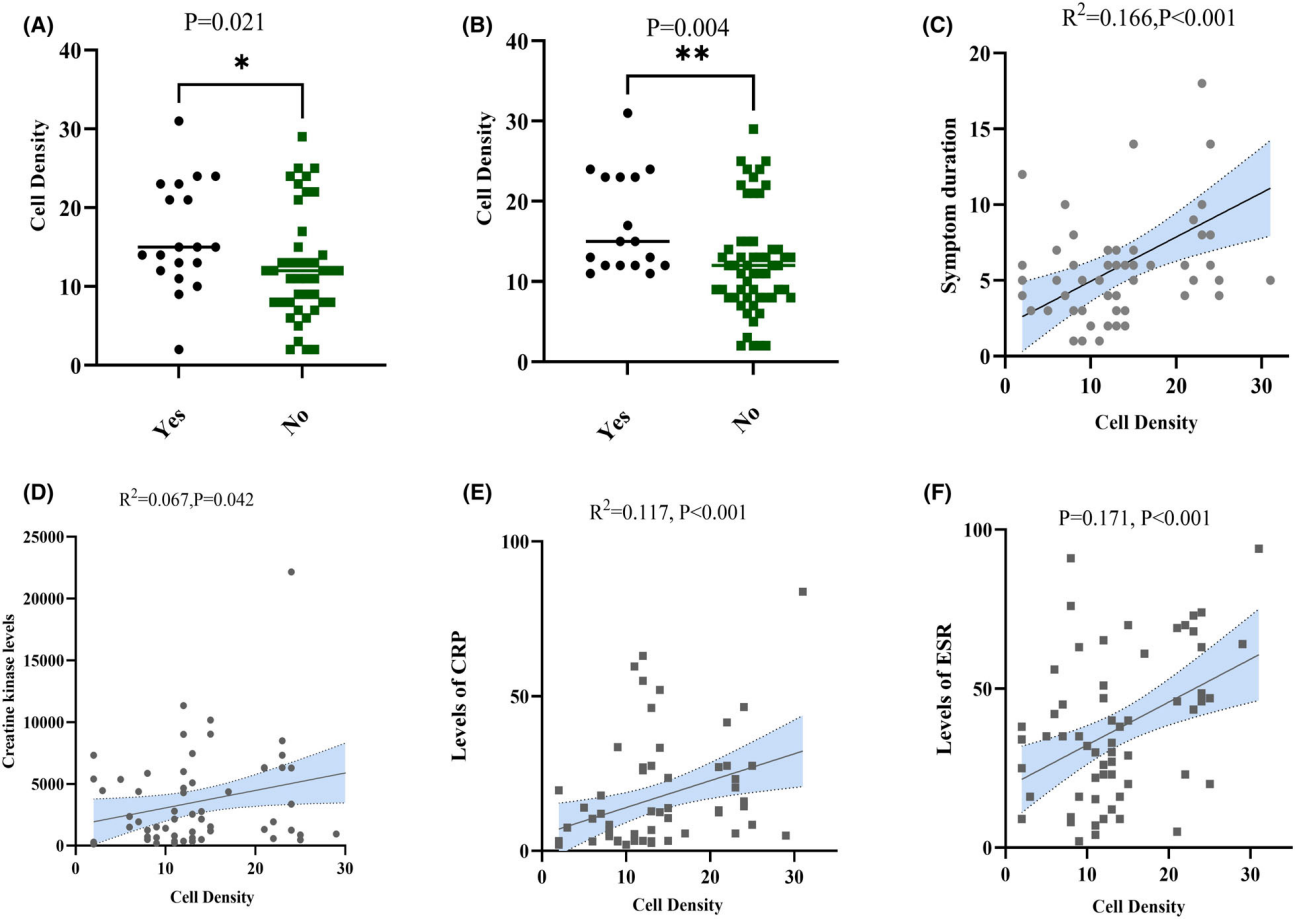
Correlation analysis of CD163+ perimysial connective tissue densities with clinical parameters. (A) Cardiac involvement. (B) Dysphagia. (C) Symptom duration. (D) Creatine kinase. (E) C‐reactive protein (CRP). (F) Erythrocyte sedimentation rate (ESR).

Regardless of the cluster distribution of IMNM patients, we correlated the density of CD163+ macrophages in perimysial connective tissue with relevant clinical parameters. Significant variables identified in the study included cardiac involvement (*p* = 0.021), dysphagia (*p* = 0.004), symptom duration (*R* ^2^ = 0.166, *p* < 0.001), CK (*R* ^2^ = 0.067, *p* = 0.042), CRP (*R* ^2^ = 0.117, *p* < 0.001), and ESR (*R* ^2^ = 0.171, *p* < 0.001) levels (Fig. 5).

## Discussion

This is the first study to investigate the relationship between macrophage markers and prognosis in IMNM patients on a large sample size. The study results revealed that: (1) The predominant macrophage infiltration subtypes in IMNM include CD206+ and CD163+ macrophages; (2) the density of CD163+ macrophages in perimysial connective tissue emerged as an independent risk factor for unfavorable prognosis; (3) unsupervised cluster analysis allowed patients to be divided into three clusters with different clinical characteristics and outcomes.

The perimysial connective tissue of the samples showed a strong correlation between macrophage levels of CD163+ and unfavorable outcomes. During follow‐up periods, 35.5% of IMNM patients reached the unfavorable endpoint. Cluster 3 exhibited an enrichment of CD163+ macrophages compared to other clusters, emphasizing the need to develop targets to intervene in macrophage function. In different clusters, symptom duration, severe myasthenia, cardiac involvement, serum inflammatory marker levels (ESR, CRP, C4), and CD163+ macrophage distribution were confirmed as discriminators. Cluster 1 patients exhibited a relatively brief disease duration, mild clinical and pathological manifestations, and consistently low levels of inflammation across all three clusters, indicating a state of low immunoinflammatory burden. Based on these findings, patients in Cluster 3 were more likely to experience severe myasthenia, cardiac involvement, and unfavorable prognosis. Allenbach and Lia et al. et al. found that the infiltration of macrophages into the perivascular endomysium influences complement deposition, leading to myonecrosis and regeneration. This process contributes to the development of a more severe clinical phenotype.^13^, ^18^ Cardiac involvement is a frequent complication of IMNM.^19^ Autopsy findings reveal massive macrophage infiltration in both skeletal and cardiac muscle.^20^ These results are consistent with previous findings that rates of ILD, dysphagia, cardiac involvement, and severe weakness increase with disease severity.^19^, ^21^, ^22^ Despite the impact of current macrophage markers on cardiac involvement is unclear, with persistent inflammation or cytokine storms as a plausible explanation.^23^

Tissue‐resident macrophages trigger two distinct responses through different metabolic programs: the destruction of infected tissue (inflammatory response, M1 macrophages) or the repair of damaged tissue (regenerative response, M2 macrophages).^24^ Conversely, M2‐type macrophages inhibit M1 macrophages, which predominantly express iNOS, by upregulating the arginine pathway while promoting tissue repair.^25^ In our study, an enrichment of M1 macrophages was observed in Cluster 1, represented by a short symptom duration, and enrichment of M2 macrophages was observed in Cluster 3, represented by a longer symptom duration. iNOS+ macrophages are mainly involved in the phagocytosis of necrotic fibers, which is consistent with the study of Allenbach et al.^18^ The density of CD163+ in connective tissue was statistically correlated with the symptom duration. This phenomenon might be referenced to the findings of SLE: M1 macrophages are initially recruited to the target organ to clear apoptotic or damaged cells, followed by a gradual shift toward M2 macrophages to maintain chronic inflammation.^9^ In addition, the CD4, CD8, and CD20 lymphocyte densities we observed were similar to earlier studies: Scattered endomysial lymphocyte infiltration was not uncommon, in addition to the routine presence of necrotic fibers and macrophages.^18^, ^26^ Due to the degree of necrosis correlated with lymphocytes, which is thought to be secondary to an immune imbalance, this imbalance does not differ between the different antibody subtypes of IMNM.^2^

CD163, known as the haptoglobin‐hemoglobin receptor, is a 130 kDa membrane protein expressed exclusively on monocytes or macrophages that binds and scavenges oxidized and pro‐inflammatory hemoglobin in autoimmune diseases.^27^, ^28^ Simultaneously, reducing pro‐inflammatory hemoglobin increases the levels of hemoglobin‐oxygenase‐1 and anti‐inflammatory hemoglobin metabolites, resulting in significant anti‐inflammatory effects.^28^, ^29^ CD163 receptor upregulation is a significant risk marker for patient survival in both acute and chronic inflammation and represents a crucial macrophage activation phenotype.^28^ Tae Chung et al. analyzed immune cells from 18 cases of HMGCR‐IMNM. All cases showed an increase in intramyocardial or perivascular CD163+ M2 macrophages, while CD11c + M1 macrophages accounted for only 18.8%.^30^ Furthermore, the authors hypothesized that M2 subtype macrophages promote muscle regeneration rather than mediate muscle damage.^30^ In individuals with polymyositis and dermatomyositis, serum CD163 levels were positively correlated with disease activity and macrophage density in muscle tissue.^31^, ^32^ The findings are consistent with our findings that the upregulation of CD163 expression in IMNM patients is associated with more severe immune inflammation and treatment resistance. However, it is unclear whether CD163+ macrophages are involved in disease progression or are recruited to prevent the progressive inflammatory process. Further studies are needed to investigate the presence of free CD163+ proteins in the serum of IMNM patients to explore their predictive value for relapse or poor recovery.

Semiquantitative analysis of tissue characteristics based on IHC is a reproducible method that can be quickly implemented in clinical practice. Our study quantified macrophage density into three measures: perivascular, endomysial, and perimysial connective tissue infiltration. The results showed that CD163+ macrophage infiltration in perimysial connective tissue was an independent risk factor for unfavorable response to immunotherapy. On the one hand, this was probably because macrophages have different biological functions at different locations. For example, in tumors, tumor‐associated macrophages promote tumor invasion and induce angiogenesis in the primary tumor. After metastasis, macrophages sustain continued growth by promoting extravasation and tumor survival.^33^ It is worth noting that CD163+ macrophages in the perimysial connective tissue may have a more significant function than CD163+ macrophages in the perivascular and endomysial regions. On the other hand, connective tissue has the highest density of CD163+ macrophages of all histologic regions, making it the most susceptible to immune inflammation. Immunoinflammation can lead to damage by activating nuclear factor κB (NFκB) signaling and increase the production of pro‐inflammatory cytokines such as IL‐1, IL‐6, and TNF‐α.^34^, ^35^ M2‐type macrophages that are imbalanced produce CCL18, which can lead to excessive tissue repair. This, in turn, promotes fibroblast proliferation and collagen deposition.^35^ Therapies that target macrophages can be classified as those that target macrophage receptors, such as blocking the “eat me” signal from CD47, or those that indirectly target cytokines secreted by or acting on macrophages.^36^ In addition, as CD163+ macrophages are upregulated in inflammation, the CD163 receptor can be targeted via antibody‐drug coupling resulting in triggering endocytosis and release of the drug to fulfill its function.^36^ Targeted dexamethasone is an excellent example of drug delivery,^37^ which requires realistic animal models of IMNM for multiple validations.

This study has several important limitations. First, the classification of macrophage subtypes based on membrane surface markers has restrictions. A minority of macrophages may express overlapping markers rather than stable extremes. Second, simple sample sizes and single‐center nature may affect statistical power owing to the low prevalence of IMNM. Finally, the retrospective design and heterogeneity of immunotherapy restrict the predictive value of the study. External validation and prospective research are needed to confirm and clarify the potential role of CD163+ macrophage density in disease management and prognosis.

## Conclusion

The study found that the density of CD163+ macrophages in perimysial connective tissues was identified as an independent risk factor for poor prognosis in IMNM patients. Patients can be classified into three various clusters using unsupervised cluster analysis, and a decision tree was contributed to quickly identify patient risk labels. The findings offered novel insights into the prognostic markers and target interventions in IMNM patients.

## Author Contributions

H.S. and X.Y. participated in the study design and drafting of the manuscript. Z.W. assisted with language review and revision. H.S. and Y.H. participated in data collection and statistical analysis. H.S., Y.W., and X.W. participated in the literature search of the manuscript. All authors revised the manuscript and approved the final article.

## Funding Information

The work was supported by the Medical and Health Talents Special Foundation of Jilin Province (No. JLSWSRCZX2023‐13).

## Conflict of Interest

The authors declare the absence of any commercial or financial relationship which might constitute a potential conflict of interest.

## Supporting information


**Table S1.** Variables included in the cluster analysis.
**Table S2.** Variables included in the multivariate logistic regression.
**Table S3.** Validation cohort in the decision tree.

**Figure S1.** Example of calculation of mean macrophage cell density. The Qupath software should be utilized to outline the regions of interest labeled 1, 2, and 3. The data within the same label was averaged in a single field of view. For instance, to determine the density of CD163+ macrophages in perimysial connective tissue (purple area, labeled 3), count the number of DAB‐positive cells per unit area. Perivascular was defined as positive cells within the blood vessels or attached to the outer wall of the blood vessels.

**Figure S2.** Heatmap of *z*‐score [*z*‐score = (*xi* − mean(*x*))/SD(*x*)] for each cluster and sample.

**Figure S3.** Variable importance scores for predictors in the decision tree. The “caret” R package enabled variable normalization, scoring them based on their relative reduction in the loss function. Additionally, the package listed important candidate variables that were not used in the segmentation due to limitations in sample size.

**Figure S4.** Decision tree modeling of receiver operating curves for development cohort.

## Data Availability

All raw data are available from the corresponding author without any reservation.
