# Supplementary material for: CD163+ macrophage density in perimysial connective tissue associated with prognosis in IMNM
Source: Ann Clin Transl Neurol. 2024 Apr 23;11(5):1267–79. doi: 10.1002/acn3.52065 (PMC11093240; doi:10.1002/acn3.52065)
Supplement: Supplementary file 1 — Table S1. Variables included in the cluster analysis. Table S2. Variables included in the multivariate logistic regression. Table S3. Validation cohort in the decision tree. [file ACN3-11-1267-s002.docx]

Supplementary Materials

**This file includes:**

Supplementary Figure legends 1 to 5

Supplementary Tables 1 to 3

*Correspondence e-mail: [xuefan@jlu.edu.cn](mailto:xuefan@jlu.edu.cn)

| Counts by pathological features | Variable type |
| --- | --- |
| iNOS+ endomysial infiltration | continuous |
| iNOS+ perivascular infiltration | continuous |
| iNOS+ perimysial connective tissue | continuous |
| CD206+endomysial infiltration | continuous |
| CD206+perivascular infiltration | continuous |
| CD206+ perimysial connective tissue | continuous |
| CD163+ endomysial infiltration | continuous |
| CD163+ perivascular infiltration | continuous |
| CD163+ perimysial connective tissue | continuous |
| Mean visual field counts (x200) |  |
| CD3 | continuous |
| CD4 | continuous |
| CD8 | continuous |
| CD20 | continuous |
| CD68 | continuous |
| Myofibre necrosis | continuous |
| Myofibre regeneration | continuous |
| Myofibre C5b-9 deposition | Categorical (0 = absence, 1 = isolated/sporadic and/or 1-3 per 20x field, 2 = scattered (4 or more) per 20x field) |
| Vascular C5b-9 deposition |  |

**Supplementary table 1. Variables included in the cluster analysis.**

**Supplementary table 2. Variables included in the multivariate logistic regression.**

| Variables | OR (95%CI) | OR | P-value |
| --- | --- | --- | --- |
| iNOS+ perimysial connective tissue infiltration | 0.800,1.789 | 1.197 | 0.382 |
| iNOS+ endomysial infiltration | 0.726,1.574 | 1.069  1.359 | 0.734 |
| CD206+ perivascular infiltration  CD206+ perimysial connective tissue | 0.343,5.391 | 1.359 | 0.663 |
| CD206+ perimysial connective tissue infiltration | 0.518,1.005 | 0.722  1.248 | 0.054 |
| CD163+ endomysial infiltration  CD163+ perivascular | 0.912,1,707 | 1.248  2.041 | 0.166 |
| CD163+ perivascular infiltration | 0.676,6.157 | 2.041  1.463 | 0.205 |
| CD163+ perimysial connective tissue infiltration | 1.049,2.041 | 1.463 | 0.025 |
| Symptom duration | 0.803,1.612 | 1.138 | 0.467 |
| Dysphagia | 0.068,32.239 | 1.483 | 0.802 |

**Supplementary table 3. Validation cohort in the Decision Tree.**

| Variables | Number(n=11) |
| --- | --- |
| Low density CD163+ in perimysial connective tissue | 9 |
| Symptom duration < 5 months | 6 |
| Cluster 1 | 4 |
| Cluster 2 | 5 |
| Cluster 3 | 2 |

Accuracy (72.7%) = Actual classification (n=8) / Predicted classification (n=11)
