# Supplementary figures and images for: CD163+ macrophage density in perimysial connective tissue associated with prognosis in IMNM
Source: Ann Clin Transl Neurol. 2024 Apr 23;11(5):1267–79. doi: 10.1002/acn3.52065 (PMC11093240; doi:10.1002/acn3.52065)

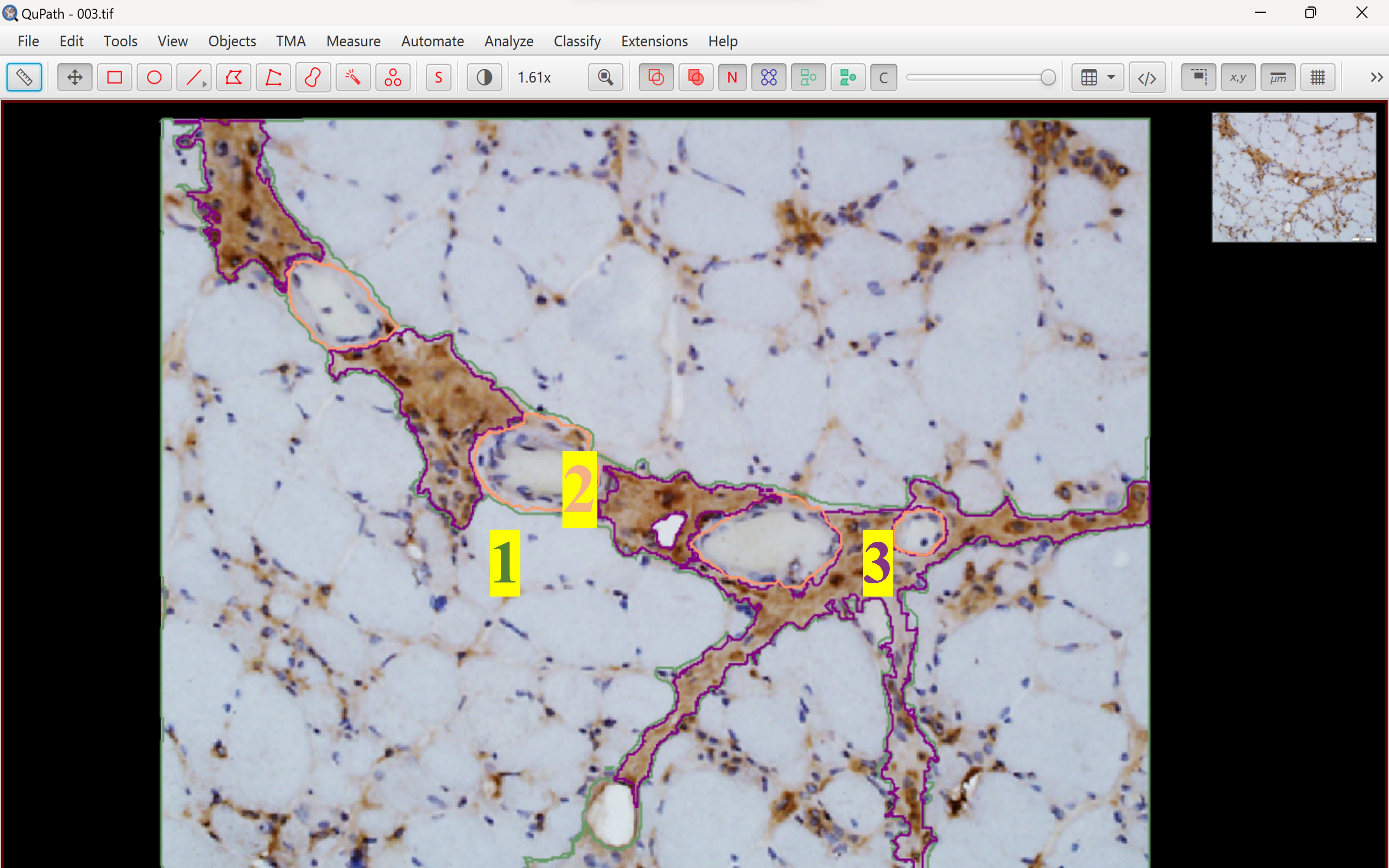

Supplement: Supplementary file 2 — Figure S1. Example of calculation of mean macrophage cell density. The Qupath software should be utilized to outline the regions of interest labeled 1, 2, and 3. The data within the same label was averaged in a single field of view. For instance, to determine the density of CD163+ macrophages in perimysial connective tissue (purple area, labeled 3), count the number of DAB‐positive cells per unit area. Perivascular was defined as positive cells within the blood vessels or attached to the outer wall of the blood vessels. [file ACN3-11-1267-s005.jpg]

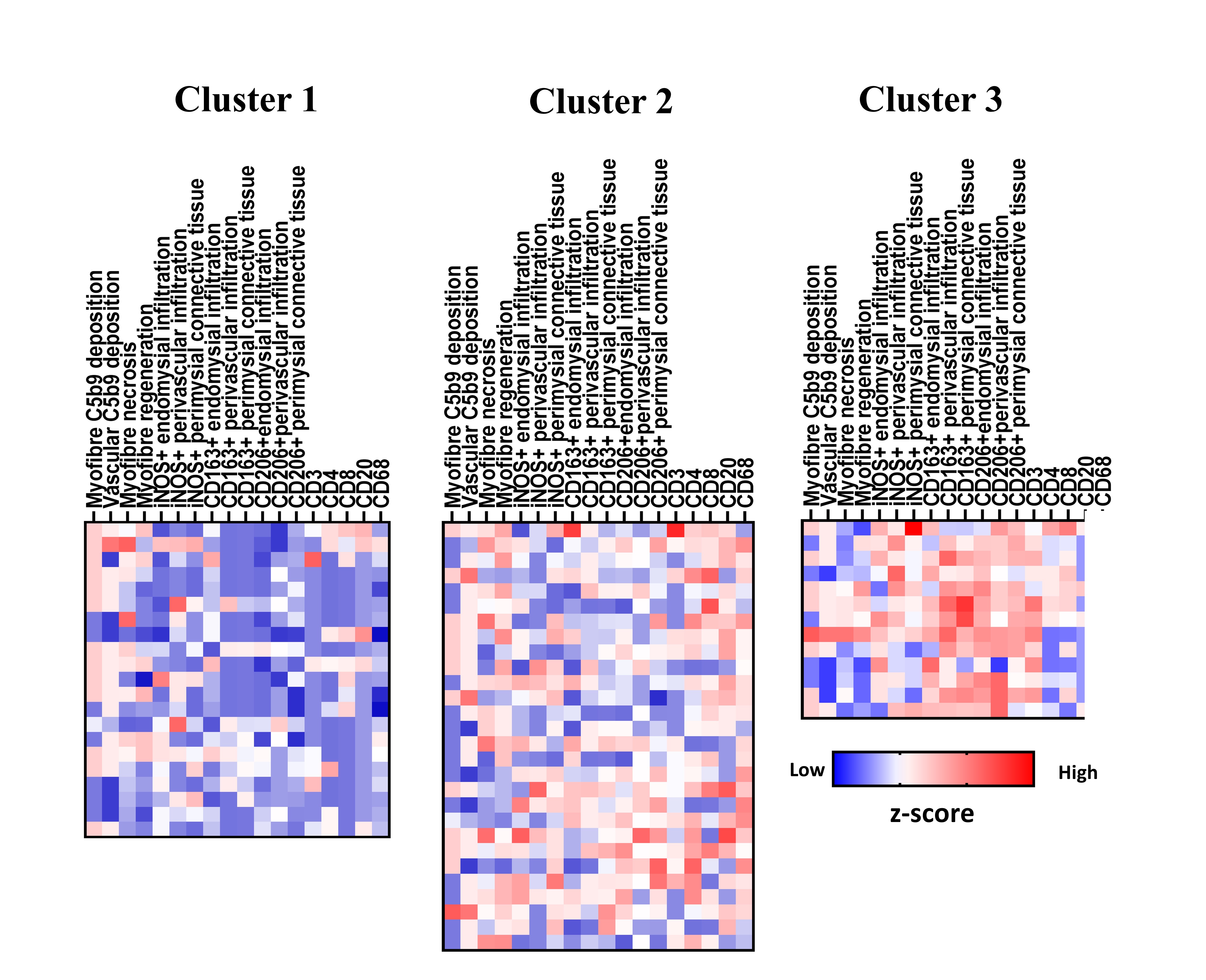

Supplement: Supplementary file 3 — Figure S2. Heatmap of z‐score [z‐score = (xi − mean(x))/SD(x)] for each cluster and sample. [file ACN3-11-1267-s003.jpg]

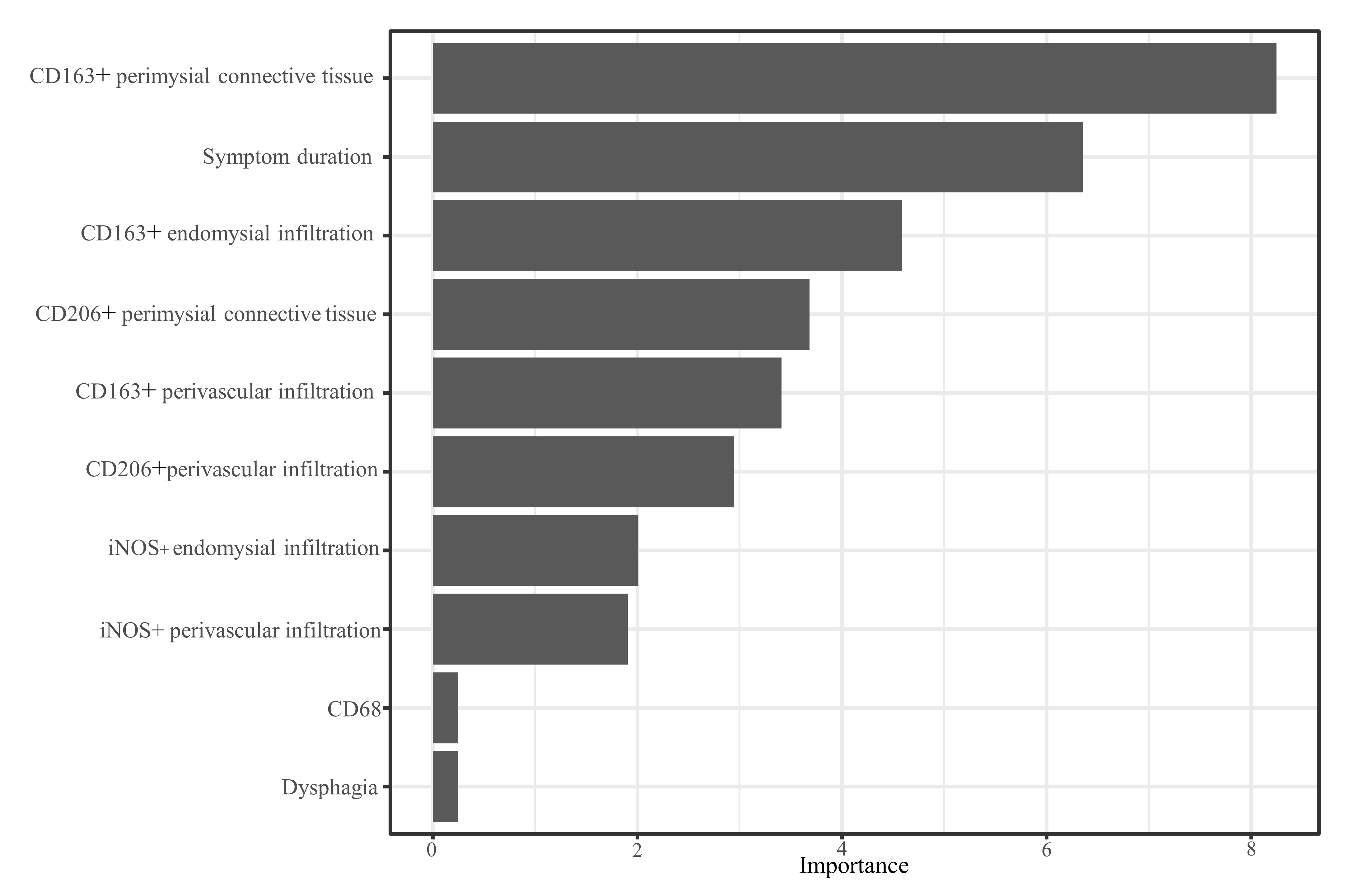

Supplement: Supplementary file 4 — Figure S3. Variable importance scores for predictors in the decision tree. The “caret” R package enabled variable normalization, scoring them based on their relative reduction in the loss function. Additionally, the package listed important candidate variables that were not used in the segmentation due to limitations in sample size. [file ACN3-11-1267-s004.jpg]

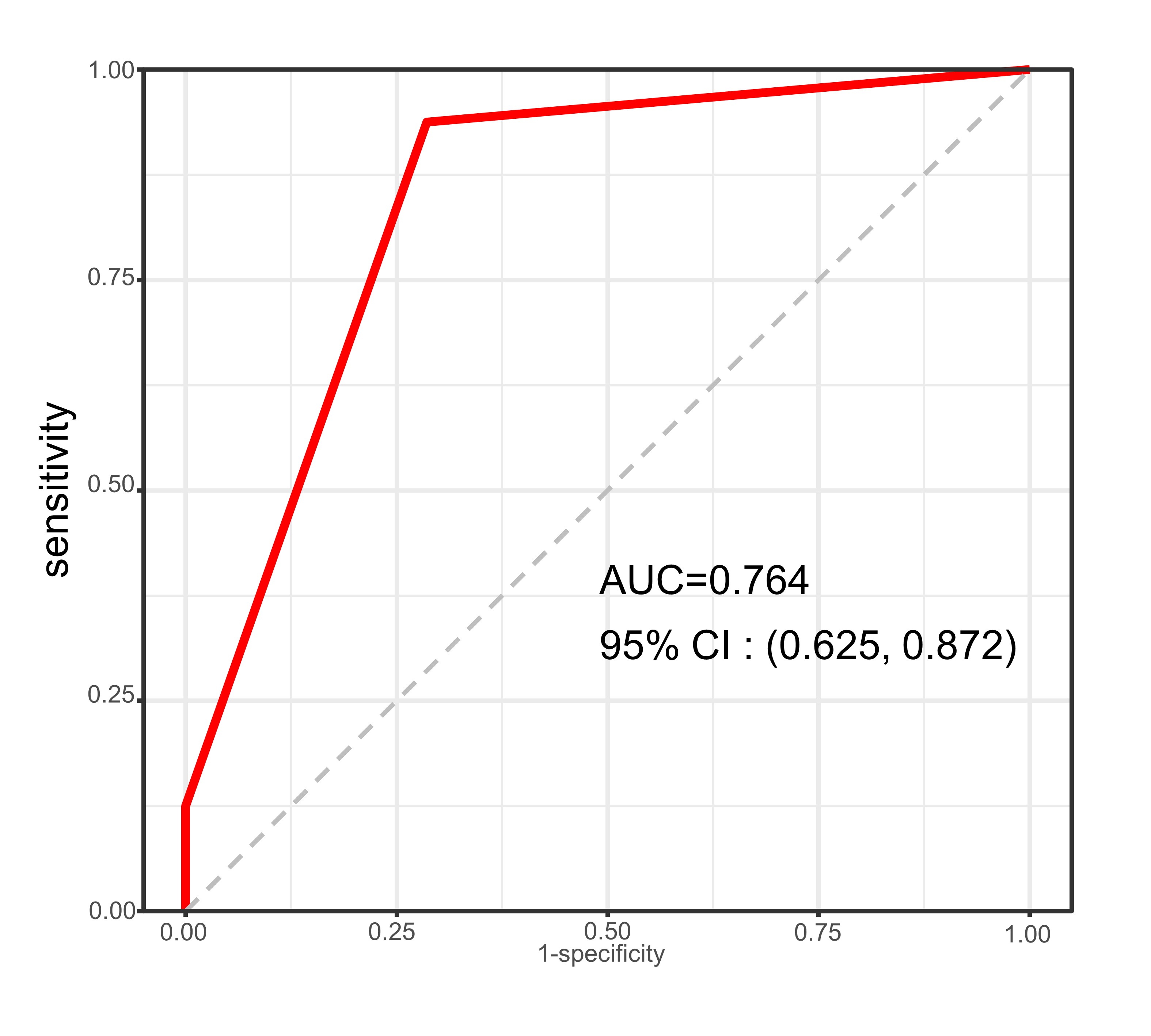

Supplement: Supplementary file 5 — Figure S4. Decision tree modeling of receiver operating curves for development cohort. [file ACN3-11-1267-s001.jpg]
